# 1-(2-Furo­yl)-3-(1-naphth­yl)thio­urea

**DOI:** 10.1107/S1600536808012208

**Published:** 2008-05-14

**Authors:** J. Duque, Osvaldo Estevez-Hernandez, Edilso Reguera, Rodrigo S. Corrêa, P. Gutierrez Maria

**Affiliations:** a Department of Structure Analysis, Institute of Materials, University of Havana, Cuba; b Grupo de Cristalografía, Instituto de Física de São Carlos, Universidade de São Paulo, São Carlos, Brazil; c Institute of Materials, UNAM, Av. Universidad No 3000 Col. Copilco el Alto, DF, Mexico

## Abstract

In the title compound, C_16_H_12_N_2_O_2_S, the carbonyl­thio­urea group forms dihedral angles of 75.4 (1) and 13.1 (2)°, respectively, with the naphthalene ring system and furan ring. The mol­ecule adopts a *trans*–*cis* configuration with respect to the positions of the furoyl and naphthyl groups relative to the S atom across the thio­urea C—N bonds. This geometry is stabilized by an N—H⋯·O intra­molecular hydrogen bond. In the crystal structure, mol­ecules are linked by N—H⋯S hydrogen bonds, forming centrosymmetric dimers which are inter­linked through C—H⋯π inter­actions.

## Related literature

For general background, see: Ashraf *et al.* (2007 ▶); Koch (2001 ▶). For related structures, see: Dago *et al.* (1987 ▶); Cao *et al.* (1996 ▶); Yuan *et al.* (1997 ▶); Kaminsky *et al.* (2002 ▶); Weiqun *et al.* (2003 ▶); Yamin & Hassan (2004 ▶). For the synthesis, see: Otazo *et al.* (2001 ▶).
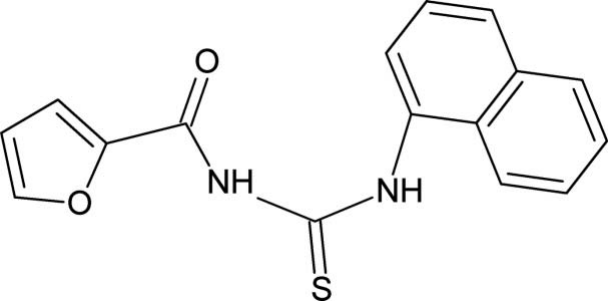


## Experimental

### 

#### Crystal data

C_16_H_12_N_2_O_2_S
*M* *_r_* = 296.34
Monoclinic, 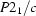

*a* = 9.402 (2) Å
*b* = 19.082 (4) Å
*c* = 7.880 (2) Å
β = 94.94 (1)°
*V* = 1408.5 (6) Å^3^
*Z* = 4
Mo *K*α radiation
μ = 0.23 mm^−1^
*T* = 294 (2) K
0.50 × 0.25 × 0.05 mm

#### Data collection

Siemens P4 diffractometer
Absorption correction: none
3603 measured reflections
2771 independent reflections
1521 reflections with *I* > 2σ(*I*)
*R*_int_ = 0.043
3 standard reflections every 97 reflections intensity decay: 2.6%

#### Refinement

*R*[*F* ^2^ > 2σ(*F* ^2^)] = 0.058
*wR*(*F* ^2^) = 0.131
*S* = 1.02
2771 reflections
239 parameters
All H-atom parameters refined
Δρ_max_ = 0.37 e Å^−3^
Δρ_min_ = −0.22 e Å^−3^

### 

Data collection: *XSCANS* (Siemens, 1996 ▶); cell refinement: *XSCANS*; data reduction: *XSCANS*; program(s) used to solve structure: *SHELXS97* (Sheldrick, 2008 ▶); program(s) used to refine structure: *SHELXL97* (Sheldrick, 2008 ▶); molecular graphics: *ORTEP-3 for Windows* (Farrugia, 1997 ▶); software used to prepare material for publication: *WinGX* (Farrugia, 1999 ▶).

## Supplementary Material

Crystal structure: contains datablocks global, I. DOI: 10.1107/S1600536808012208/ci2591sup1.cif

Structure factors: contains datablocks I. DOI: 10.1107/S1600536808012208/ci2591Isup2.hkl

Additional supplementary materials:  crystallographic information; 3D view; checkCIF report

## Figures and Tables

**Table 1:** Hydrogen-bond geometry (Å, °)

*D*—H⋯*A*	*D*—H	H⋯*A*	*D*⋯*A*	*D*—H⋯*A*
N2—H2⋯O1	0.86 (4)	2.00 (4)	2.698 (3)	138 (3)
N1—H1⋯S1^i^	0.91 (5)	2.57 (5)	3.455 (3)	164 (4)
C5—H5⋯*Cg*1^ii^	0.96 (4)	2.85 (4)	3.654 (4)	143 (3)

Symmetry codes: (i) 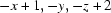; (ii) 
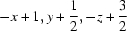
. *Cg*1 is the centroid of the C7–C11/C16 ring.

**Fig. 1.**
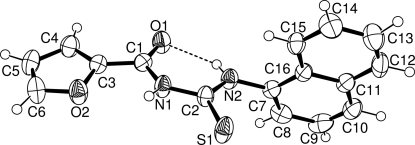
The molecular structure of the title compound. Displacement ellipsoids are drawn at the 50% probability level. The intramolecular N—H···O hydrogen bond is shown as a dashed line.

**Fig. 2.**
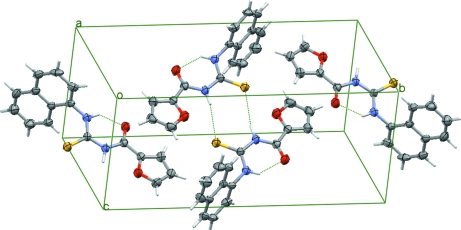
View of the crystal packing of the title compound. Hydrogen bonds are shown as dashed lines.

## supplementary crystallographic information

### Comment

The subject of aroylsubstituted thioureas is considered as a very interesting topic due to their remarkable optical and electronic properties (Ashraf *et al.*,2007). Substitutions that reduce the symmetry of the thiourea molecule enhance the non-linear optical properties. A variety of crystals of this class has been reported (Dago *et al.*, 1987; Cao *et al.*, 1996; Yuan *et al.*, 1997; Kaminsky *et al.*, 2002; Weiqun *et al.*, 2003). The title compound (Fig.1) is another example of a newly synthesized furoylthiourea derivative.

The bond lengths and angles are comparable with those observed in other thiourea derivatives (Koch *et al.*, 2001). The α-naphtalene ring system attached to N2 is essentially planar and inclined at an angle of 75.4 (1)° with respect to the plane of carbonylthiourea group. The dihedral angle between the carbonylthiourea group and furan ring is 13.1 (2)°. The molecule adopts a trans-cis configuration with respect to the position of the furoyl and naphthyl groups relative to the S atom across the thiourea C—N bonds. This geometry is stabilized by the N2—H2···.O1 intramolecular hydrogen bond (Fig.1).

In the crystal structure, molecules are linked by N1—H1···.S1 hydrogen bonds (Table 1) forming a centrosymmetric dimer (Fig. 2). The dimers are arranged along the *c* axis. In addition, the crystal packing is stabilized by C—H···π interactions involving the C7-C11/C16 ring.

### Experimental

The title compound was synthesized according to a previous report (Otazo *et al.*, 2001), by converting furoyl choride into furoyl isothiocyanate and then condensing with α-naphtylamine. The resulting solid product was crystallized from ethanol yielding X-ray quality single crystals (m.p 186–187°). Elemental analysis for C_16_H_12_N_2_O_2_S calculated: C 64.86, H 4.05, N 9.46, S 10.81%; found: C 64.70, H 4.10, N 9.54, S 10.41%.

### Refinement

All H atoms were located by difference Fourier synthesis and refined freely.

### Crystal data

**Table:**

C_16_H_12_N_2_O_2_S	*F*_000_ = 616
*M**_r_* = 296.34	*D*_x_ = 1.397 Mg m^−^^3^
Monoclinic, *P*2_1_/*c*	Mo *K*α radiation λ = 0.71073 Å
Hall symbol: -P 2ybc	Cell parameters from 37 reflections
*a* = 9.402 (2) Å	θ = 9.9–23.4º
*b* = 19.082 (4) Å	µ = 0.24 mm^−^^1^
*c* = 7.880 (2) Å	*T* = 294 (2) K
β = 94.94 (1)º	Plate, white
*V* = 1408.5 (6) Å^3^	0.50 × 0.25 × 0.05 mm
*Z* = 4	

### Data collection

**Table:**

Siemens P4 diffractometer	θ_max_ = 26.0º
Radiation source: fine-focus sealed tube	θ_min_ = 2.1º
Monochromator: graphite	*h* = −11→11
2θ/ω scans	*k* = −23→1
Absorption correction: none	*l* = −1→9
3603 measured reflections	3 standard reflections
2771 independent reflections	every 97 reflections
1521 reflections with *I* > 2σ(*I*)	intensity decay: 2.6%
*R*_int_ = 0.043	

### Refinement

**Table:**

Refinement on *F*^2^	All H-atom parameters refined
Least-squares matrix: full	*w* = 1/[σ^2^(*F*_o_^2^) + (0.0395*P*)^2^ + 0.2114*P*] where *P* = (*F*_o_^2^ + 2*F*_c_^2^)/3
*R*[*F*^2^ > 2σ(*F*^2^)] = 0.058	(Δ/σ)_max_ < 0.001
*wR*(*F*^2^) = 0.131	Δρ_max_ = 0.37 e Å^−^^3^
*S* = 1.02	Δρ_min_ = −0.22 e Å^−^^3^
2771 reflections	Extinction correction: SHELXL97 (Sheldrick, 2008), Fc^*^=kFc[1+0.001xFc^2^λ^3^/sin(2θ)]^-1/4^
239 parameters	Extinction coefficient: none

### Special details

**Table:**

Geometry. All e.s.d.'s (except the e.s.d. in the dihedral angle between two l.s. planes) are estimated using the full covariance matrix. The cell e.s.d.'s are taken into account individually in the estimation of e.s.d.'s in distances, angles and torsion angles; correlations between e.s.d.'s in cell parameters are only used when they are defined by crystal symmetry. An approximate (isotropic) treatment of cell e.s.d.'s is used for estimating e.s.d.'s involving l.s. planes.

### Fractional atomic coordinates and isotropic or equivalent isotropic displacement parameters (Å^2^)

**Table:**

	*x*	*y*	*z*	*U*_iso_*/*U*_eq_	
S1	0.57874 (10)	−0.05278 (4)	0.80311 (14)	0.0521 (3)	
O1	0.7296 (2)	0.17234 (11)	0.7355 (3)	0.0472 (7)	
O2	0.4090 (2)	0.17986 (11)	0.9419 (3)	0.0505 (7)	
N1	0.5895 (3)	0.08508 (13)	0.8319 (4)	0.0404 (8)	
N2	0.7522 (3)	0.03510 (14)	0.6602 (4)	0.0382 (7)	
C7	0.8101 (3)	−0.02051 (16)	0.5647 (4)	0.0334 (8)	
C11	0.9652 (3)	−0.12250 (17)	0.5475 (5)	0.0378 (9)	
C2	0.6455 (3)	0.02544 (15)	0.7612 (4)	0.0355 (8)	
C8	0.7742 (4)	−0.0249 (2)	0.3936 (5)	0.0439 (9)	
C16	0.9075 (3)	−0.06863 (15)	0.6463 (4)	0.0334 (8)	
C1	0.6256 (3)	0.15409 (16)	0.8096 (4)	0.0345 (8)	
C3	0.5298 (3)	0.20467 (16)	0.8776 (4)	0.0343 (8)	
C10	0.9242 (4)	−0.1259 (2)	0.3713 (5)	0.0468 (10)	
C15	0.9520 (4)	−0.06626 (19)	0.8244 (5)	0.0399 (9)	
C6	0.3331 (4)	0.2368 (2)	0.9858 (5)	0.0538 (11)	
C5	0.4016 (4)	0.2956 (2)	0.9518 (5)	0.0492 (10)	
C4	0.5283 (4)	0.27538 (18)	0.8823 (5)	0.0453 (9)	
C12	1.0639 (4)	−0.1706 (2)	0.6295 (6)	0.0521 (11)	
C9	0.8308 (4)	−0.0782 (2)	0.2963 (6)	0.0530 (11)	
C14	1.0479 (4)	−0.1136 (2)	0.8953 (6)	0.0526 (11)	
C13	1.1034 (4)	−0.1664 (2)	0.7981 (6)	0.0564 (11)	
H15	0.918 (3)	−0.0323 (14)	0.890 (4)	0.024 (8)*	
H4	0.602 (3)	0.3026 (16)	0.844 (4)	0.040 (9)*	
H10	0.963 (3)	−0.1650 (19)	0.306 (5)	0.055 (10)*	
H12	1.106 (3)	−0.2074 (18)	0.560 (4)	0.055 (10)*	
H6	0.242 (4)	0.228 (2)	1.031 (5)	0.072 (13)*	
H8	0.709 (3)	0.0104 (16)	0.340 (4)	0.039 (9)*	
H9	0.810 (3)	−0.0785 (16)	0.182 (5)	0.039 (10)*	
H2	0.775 (4)	0.078 (2)	0.645 (5)	0.063 (12)*	
H13	1.175 (4)	−0.197 (2)	0.860 (5)	0.081 (14)*	
H14	1.073 (4)	−0.1106 (19)	1.015 (5)	0.062 (12)*	
H1	0.531 (5)	0.072 (2)	0.913 (6)	0.092 (16)*	
H5	0.374 (4)	0.342 (2)	0.977 (6)	0.090 (14)*	

### Atomic displacement parameters (Å^2^)

**Table:**

	*U*^11^	*U*^22^	*U*^33^	*U*^12^	*U*^13^	*U*^23^
S1	0.0644 (6)	0.0296 (4)	0.0675 (8)	−0.0031 (4)	0.0355 (5)	−0.0018 (5)
O1	0.0483 (14)	0.0326 (12)	0.0632 (18)	−0.0007 (10)	0.0198 (13)	0.0038 (12)
O2	0.0541 (14)	0.0330 (12)	0.067 (2)	0.0051 (11)	0.0224 (14)	0.0050 (13)
N1	0.0461 (17)	0.0264 (14)	0.051 (2)	0.0024 (13)	0.0188 (16)	0.0012 (14)
N2	0.0428 (16)	0.0303 (16)	0.0431 (19)	0.0004 (13)	0.0125 (14)	0.0014 (14)
C7	0.0360 (17)	0.0391 (18)	0.027 (2)	−0.0058 (15)	0.0110 (15)	−0.0021 (16)
C11	0.0380 (17)	0.0396 (19)	0.038 (2)	−0.0075 (15)	0.0171 (17)	−0.0070 (17)
C2	0.0413 (18)	0.0301 (17)	0.036 (2)	0.0038 (14)	0.0104 (16)	0.0026 (16)
C8	0.041 (2)	0.055 (2)	0.037 (2)	−0.0042 (18)	0.0065 (18)	0.010 (2)
C16	0.0343 (17)	0.0330 (18)	0.035 (2)	−0.0036 (14)	0.0121 (16)	−0.0021 (15)
C1	0.0386 (18)	0.0311 (17)	0.033 (2)	0.0011 (14)	0.0004 (17)	0.0025 (16)
C3	0.0398 (18)	0.0315 (17)	0.032 (2)	0.0021 (14)	0.0036 (16)	0.0020 (16)
C10	0.050 (2)	0.048 (2)	0.045 (3)	−0.0065 (19)	0.022 (2)	−0.012 (2)
C15	0.045 (2)	0.043 (2)	0.033 (2)	0.0055 (17)	0.0101 (17)	−0.0038 (18)
C6	0.057 (2)	0.052 (2)	0.055 (3)	0.017 (2)	0.021 (2)	0.003 (2)
C5	0.070 (3)	0.037 (2)	0.042 (3)	0.015 (2)	0.009 (2)	−0.0001 (19)
C4	0.054 (2)	0.036 (2)	0.047 (3)	0.0026 (17)	0.009 (2)	0.0019 (19)
C12	0.053 (2)	0.042 (2)	0.064 (3)	0.0095 (18)	0.021 (2)	−0.009 (2)
C9	0.060 (3)	0.076 (3)	0.025 (2)	−0.021 (2)	0.014 (2)	−0.012 (2)
C14	0.057 (2)	0.066 (3)	0.035 (3)	0.009 (2)	0.004 (2)	0.004 (2)
C13	0.060 (3)	0.057 (2)	0.053 (3)	0.016 (2)	0.007 (2)	0.007 (2)

### Geometric parameters (Å, °)

**Table:**

S1—C2	1.663 (3)	C1—C3	1.453 (4)
O1—C1	1.232 (4)	C3—C4	1.350 (4)
O2—C6	1.362 (4)	C10—C9	1.364 (6)
O2—C3	1.368 (4)	C10—H10	0.99 (4)
N1—C1	1.375 (4)	C15—C14	1.362 (5)
N1—C2	1.391 (4)	C15—H15	0.90 (3)
N1—H1	0.91 (4)	C6—C5	1.331 (5)
N2—C2	1.346 (4)	C6—H6	0.97 (4)
N2—C7	1.435 (4)	C5—C4	1.408 (5)
N2—H2	0.86 (4)	C5—H5	0.96 (4)
C7—C8	1.364 (5)	C4—H4	0.93 (3)
C7—C16	1.412 (4)	C12—C13	1.351 (6)
C11—C10	1.410 (5)	C12—H12	1.00 (3)
C11—C12	1.420 (5)	C9—H9	0.90 (3)
C11—C16	1.424 (4)	C14—C13	1.393 (6)
C8—C9	1.406 (5)	C14—H14	0.95 (4)
C8—H8	0.98 (3)	C13—H13	0.99 (4)
C16—C15	1.430 (5)		
			
C6—O2—C3	106.8 (3)	C9—C10—C11	120.5 (4)
C1—N1—C2	128.8 (3)	C9—C10—H10	122 (2)
C1—N1—H1	122 (3)	C11—C10—H10	118 (2)
C2—N1—H1	109 (3)	C14—C15—C16	120.6 (4)
C2—N2—C7	123.0 (3)	C14—C15—H15	119.9 (18)
C2—N2—H2	115 (2)	C16—C15—H15	119.5 (18)
C7—N2—H2	121 (3)	C5—C6—O2	110.3 (3)
C8—C7—C16	120.4 (3)	C5—C6—H6	133 (2)
C8—C7—N2	119.4 (3)	O2—C6—H6	117 (2)
C16—C7—N2	120.1 (3)	C6—C5—C4	106.7 (3)
C10—C11—C12	122.0 (3)	C6—C5—H5	127 (3)
C10—C11—C16	119.2 (3)	C4—C5—H5	126 (3)
C12—C11—C16	118.8 (3)	C3—C4—C5	107.2 (3)
N2—C2—N1	116.9 (3)	C3—C4—H4	122.5 (19)
N2—C2—S1	123.6 (2)	C5—C4—H4	130.3 (19)
N1—C2—S1	119.5 (2)	C13—C12—C11	121.5 (4)
C7—C8—C9	120.7 (4)	C13—C12—H12	119.6 (19)
C7—C8—H8	118.4 (19)	C11—C12—H12	119 (2)
C9—C8—H8	120.9 (19)	C10—C9—C8	120.4 (4)
C7—C16—C11	118.9 (3)	C10—C9—H9	120 (2)
C7—C16—C15	123.4 (3)	C8—C9—H9	119 (2)
C11—C16—C15	117.8 (3)	C15—C14—C13	121.3 (4)
O1—C1—N1	123.1 (3)	C15—C14—H14	118 (2)
O1—C1—C3	122.0 (3)	C13—C14—H14	121 (2)
N1—C1—C3	114.9 (3)	C12—C13—C14	120.0 (4)
C4—C3—O2	108.9 (3)	C12—C13—H13	125 (2)
C4—C3—C1	133.0 (3)	C14—C13—H13	115 (2)
O2—C3—C1	117.9 (3)		
			
C2—N2—C7—C8	103.8 (4)	O1—C1—C3—C4	−1.5 (6)
C2—N2—C7—C16	−78.5 (4)	N1—C1—C3—C4	179.6 (4)
C7—N2—C2—N1	−173.3 (3)	O1—C1—C3—O2	173.3 (3)
C7—N2—C2—S1	6.3 (5)	N1—C1—C3—O2	−5.6 (5)
C1—N1—C2—N2	1.9 (5)	C12—C11—C10—C9	−179.0 (3)
C1—N1—C2—S1	−177.8 (3)	C16—C11—C10—C9	0.1 (5)
C16—C7—C8—C9	1.5 (5)	C7—C16—C15—C14	−179.1 (3)
N2—C7—C8—C9	179.1 (3)	C11—C16—C15—C14	0.8 (5)
C8—C7—C16—C11	−1.2 (4)	C3—O2—C6—C5	−0.1 (4)
N2—C7—C16—C11	−178.9 (3)	O2—C6—C5—C4	0.0 (5)
C8—C7—C16—C15	178.7 (3)	O2—C3—C4—C5	−0.2 (4)
N2—C7—C16—C15	1.1 (4)	C1—C3—C4—C5	174.9 (4)
C10—C11—C16—C7	0.4 (4)	C6—C5—C4—C3	0.1 (5)
C12—C11—C16—C7	179.6 (3)	C10—C11—C12—C13	179.4 (3)
C10—C11—C16—C15	−179.5 (3)	C16—C11—C12—C13	0.3 (5)
C12—C11—C16—C15	−0.4 (4)	C11—C10—C9—C8	0.1 (5)
C2—N1—C1—O1	−9.3 (6)	C7—C8—C9—C10	−0.9 (5)
C2—N1—C1—C3	169.6 (3)	C16—C15—C14—C13	−1.2 (6)
C6—O2—C3—C4	0.2 (4)	C11—C12—C13—C14	−0.5 (6)
C6—O2—C3—C1	−175.8 (3)	C15—C14—C13—C12	1.0 (6)

### Hydrogen-bond geometry (Å, °)

**Table:**

*D*—H···*A*	*D*—H	H···*A*	*D*···*A*	*D*—H···*A*
N2—H2···O1	0.86 (4)	2.00 (4)	2.698 (3)	138 (3)
N1—H1···S1^i^	0.91 (5)	2.57 (5)	3.455 (3)	164 (4)
C5—H5···Cg1^ii^	0.96 (4)	2.85 (4)	3.654 (4)	143 (3)

Symmetry codes: (i) −*x*+1, −*y*, −*z*+2; (ii) −*x*+1, *y*+1/2, −*z*+3/2.
